# New frontiers in dinosaur exploration

**DOI:** 10.1098/rsbl.2025.0045

**Published:** 2025-04-30

**Authors:** Susannah Maidment, Richard J. Butler

**Affiliations:** ^1^ Natural History Museum, London, UK; ^2^ School of Geography, Earth and Environmental Sciences, University of Birmingham, Birmingham, England, UK

**Keywords:** taxonomy, systematics, fieldwork, challenges, opportunities

## Abstract

Two hundred years after the naming of the first dinosaur, taxonomic studies remain an important component of dinosaur research. Around 50 new dinosaurs are named each year and are discovered from across the globe. The rate of new dinosaur discovery shows no signs of slowing, but not all geographical areas and temporal windows have been equally investigated. The potential for new dinosaur discoveries in India and Africa seems particularly high, while the Carnian, when dinosaurs probably originated, and the Middle Jurassic, when the major clades diversified, offer the best opportunities to make discoveries that will fundamentally change our understanding of dinosaur evolution. A major challenge to the discovery of new dinosaurs is funding. Frontier fieldwork is sometimes viewed as too risky to fund, while basic taxonomic work is considered to lack impact. As a consequence, we risk an ‘extinction of experience’, where researchers have limited training in the basic field- and specimen-based research that underpins our discipline. Going forward, new remote sensing techniques may help to find prospective areas, while three-dimensional scanning apps on smartphones will allow us to quickly record field data. Artificial intelligence is likely to be used increasingly for computed tomography segmentation and identification of problematic fossils.

## 1. Introduction

The first dinosaur to be named was *Megalosaurus bucklandii* in 1824 [1], although the term ‘dinosaur’ would not be invented for another 18 years [2]. In the ensuing 200 years, discoveries of new dinosaurs have continued at pace [3–5]. Dinosaurs are now known from every continent, from their origins in the Late Triassic (e.g. [6]) to the extinction of all non-avian dinosaurs at the end of the Cretaceous (e.g. [7]). For 140 million years, throughout the Jurassic and Cretaceous, dinosaurs were the dominant vertebrates in all terrestrial ecosystems. Our understanding of this diversity is only possible because of exploratory fieldwork (field prospecting and excavation) and specimen-based taxonomic and systematic research [8–10]. Here, we review the state of dinosaur taxonomy and systematics today, the prospects for discovery of new taxa through fieldwork and new taxonomic approaches, and where, both in time and space, we are most likely to find new taxa that will significantly add to our understanding of the evolution and radiation of the dinosaurs.

## 2. Taxonomy and systematics

Taxonomy, the classification of organisms, and systematics, how they are related to each other, are fundamental to evolution and biology [11]. Taxonomy and systematics provide the foundation on which all further study is built: we cannot understand how organisms change over time to evolve into new species, the rates at which these changes occur, how they occupy ecological space or the impact of major functional changes, without first understanding how many species there are and how they are related to each other [11–13]. If we seek to understand the world of the Mesozoic, how biotic and abiotic interactions shaped biodiversity through speciation and extinction over time, and whether we can establish general rules about life on the Earth that might help us to predict future changes to our biosphere, then the discovery of new species, either through the finding of new specimens in the field or through reappraisal of specimens in museum collections, is the most critical sort of work that we do.

## 3. The state of dinosaur taxonomy today

Taxonomic work remains an important component of modern dinosaur research, with 514 taxonomic publications (approx. 50 annually) recorded in the publications database Scopus from 2014 to 2023 (see ‘Methods’ for details). However, while the total number of publications matching the term ‘dinosaur’ has been broadly static over the last decade (mean per year: approx. 347), the number of publications including taxonomic elements appears to have been declining over the last decade (from approx. 20% of total publications in 2014 to approx. 11% in 2023), apparently driven primarily by declines in numbers by authors from the United States (there are no clear trends apparent in the number of publications for authors from other key contributing countries). The proportion of dinosaur taxonomy publications in ‘top journals’ (defined by Scopus using CiteScore percentile) is approximately 15%, well below that for dinosaur publications as a whole (approx. 34%), which could explain a move away from taxonomic work (particularly that not focused on descriptions of new species) by some palaeontologists, due to institutional pressure to produce ‘high impact’ work. Taxonomic work on dinosaurs is dominated by researchers from the United States, followed in order of relative contribution by the United Kingdom, Argentina, Canada, China, Spain, Germany and Brazil, reflecting the size, resources and rich histories of dinosaur discovery in these countries.

**Figure 1.**
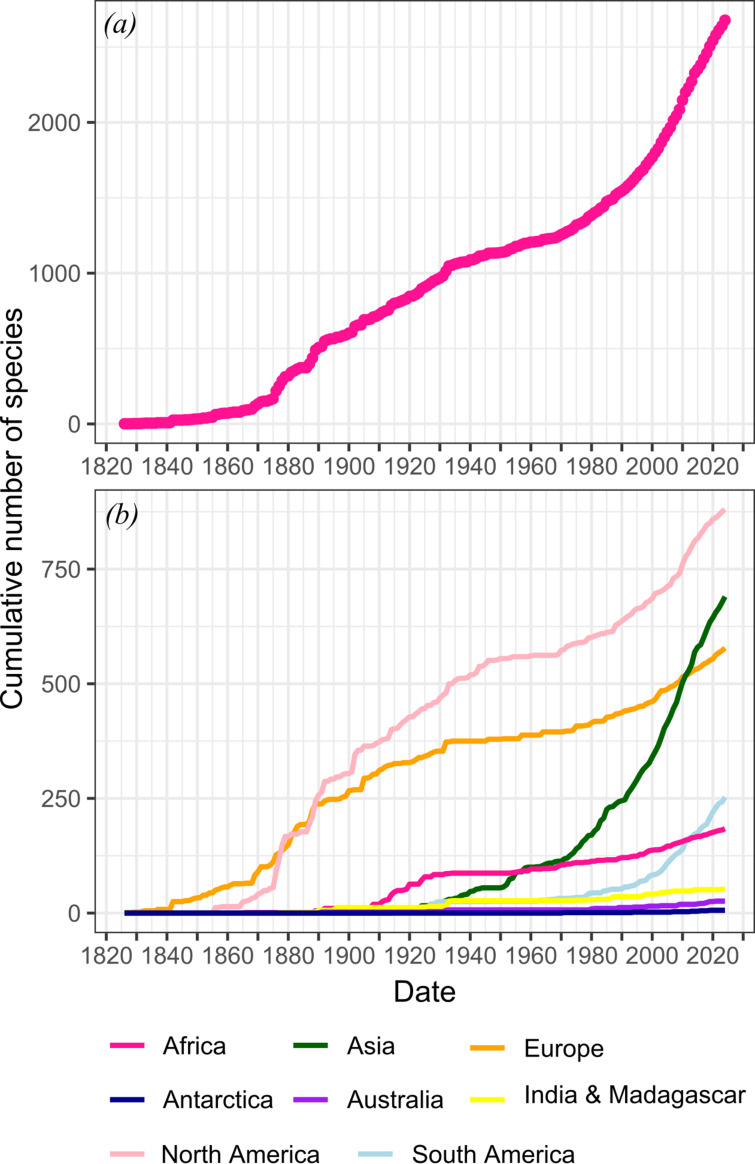
Collector curves showing the number of dinosaur species published through time. (*a*) Total number of species published and (*b*) numbers of species by continental area.

As of mid-December 2024, 2677 non-avialan dinosaur species had been named, of which 1383 are considered valid today (excluding ichno- and ootaxa). The rate of the naming of new species has broadly increased over time (figure 1*a*; [5,8]; see ‘Methods’ for details). From the 1820s to the late 1870s, the description of new species was slow. The rate of discovery increased significantly from 1877 until about 1890, however, initiated by the discovery of the dinosaurs of the Morrison Formation of the American West and the ‘bone wars’ [14,15]. The rate of discovery was steady in the subsequent half-century, before slowing in the early 1930s and remaining low until the late 1960s. The rate increase of the modern era started in the late 1960s and early 1970s (the ‘dinosaur renaissance’ [16]) and reached the highest rate between about 2009 and 2014. The slight decline in rate subsequent to 2014 may correlate with the decline in taxonomic publications over the last decade observed in Scopus data above.

Rates of discovery of new dinosaur taxa have varied geographically, as well as temporally (figure 1*b*). Rapid rates from the 1870s to 1930s were primarily driven by discoveries in Europe and North America, with Africa also contributing between 1910 and 1930 [17–19]. In contrast, rapid rates of discovery have occurred in Asia and, to a lesser extent, in South America over the past 50 years [19,20]. Asymptotes have not been reached on any continent, indicating that there are still dinosaurs being found on all, and rates of discovery have increased on every continent since the 1970s. The lowest rates of discovery during the twenty-first century have occurred in India and Madagascar, Australia, Antarctica and, to a lesser extent, Africa.

Variations in the recovery rate across different continents can probably be attributed primarily to the concentration of palaeontologists working in those regions [8]. The earliest palaeontologists were naturalists from Europe who had the time, resources, education and contacts to spend time collecting, studying and interpreting fossils [21,22]. North American scientists soon followed suit, and the building of infrastructure such as railways across the American West in the mid-1800s led to the discovery of vast numbers of dinosaur fossils and the start of the ‘bone wars’ [14,15]. Early discoveries in Africa were driven by colonial exploration, primarily by the Germans and British [17–19]. The late twentieth century rise in rates of discovery, first in Asia and then South America, can be attributed to important dinosaur discoveries in China and Argentina beginning in the latter half of the century [19,20], and today there may well be more palaeontologists working in these continents than there are in Europe. However, political conditions, climate, geology and land area also play a role in variations in discovery rates across continents. Dinosaur fossils are most likely to be found where terrestrial Mesozoic rocks crop out at the surface, and where they are well exposed, so they can be most easily found in arid and semi-arid environments. Wars and other geopolitical conflicts can prevent fieldwork taking place, and political climates unfavourable to science can prevent hiring of palaeontologists and reduce funding for their activities (e.g. [23]).

## 4. What geographical areas represent the ‘frontier’ for new dinosaur exploration?

**Figure 2.**
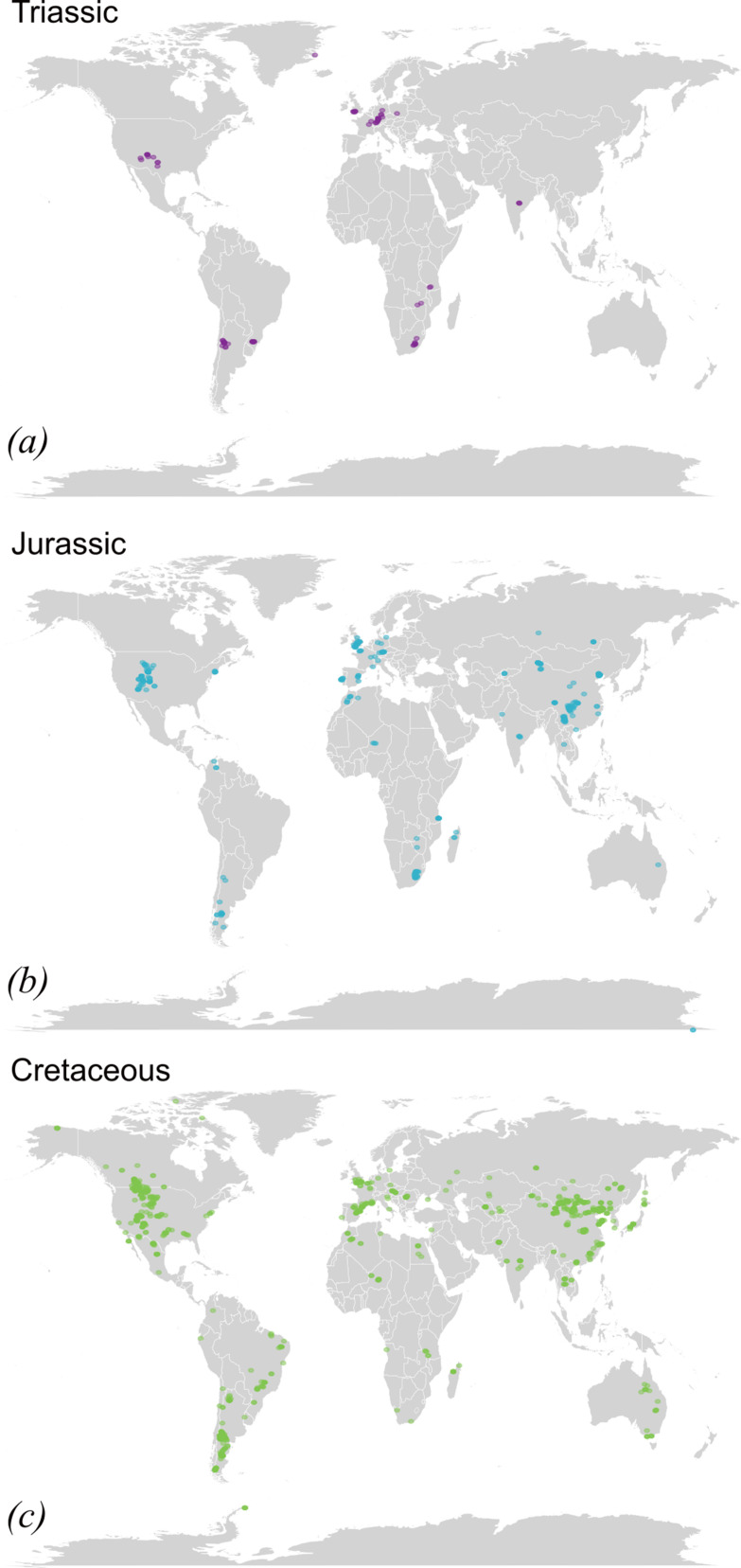
The geographical distribution of species holotypes in (*a*) the Triassic, (*b*) the Jurassic and (*c*) the Cretaceous periods. Coloured dots represent individual species’ holotypes.

The parts of the world with the fewest known dinosaur fossils are Antarctica, Australia, India and Madagascar (figures 1*b* and 2), an enormous combined landmass representing parts of the former supercontinent of Gondwana. Just six dinosaur taxa have been described from Antarctica, although there is at least one more that is currently undescribed [24], and there are fragmentary, indeterminate specimens that might represent others [25,26]. Early Jurassic terrestrial sediments crop out in the Transantarctic Mountains [27], while Late Cretaceous rocks crop out on the margins of the continent and, although these represent shallow marine rather than terrestrial environments, recent research has demonstrated the potential for new discoveries [26]. Antarctica’s ice sheets are melting at an unprecedented rate [28], exposing rock unexplored by palaeontologists, and it is possible that unknown Mesozoic ecosystems await discovery on the continent. However, fieldwork in Antarctica is logistically challenging and extremely expensive.

Twenty-six dinosaur taxa have been described to date in Australia, although the diversity is considered likely to have been higher [29]. There are extensive Cretaceous outcrops across the continent [30,31], a long history of discovery and description [30,32,33] and thousands of fossil plants, invertebrates and vertebrates are known [33], so it is possible that the relatively sparse dinosaur fossil record is, at least in part, a genuine pattern. The southeastern part of Australia was located at latitudes of greater than 70° south during the Cretaceous [30,34], and animals that lived there would have had to contend with a climate that was highly seasonal with periods of extreme cold and extended winter darkness [29]. Indeed, it has been hypothesized that cooling in the mid-Cretaceous could have prevented the dispersal of sauropods into the continent via Antarctica until the climate ameliorated in the Late Cretaceous [34].

The earliest dinosaur discoveries in India and Madagascar occurred in the late 1800s, yet only 52 species have been named, and only 22 are considered valid today. India’s Mesozoic rock record includes Late Triassic, Early and Middle Jurassic and Late Cretaceous dinosaur-bearing strata, but many of the outcrops are under-explored, and finds are fragmentary [35]. Madagascar also has outcrops of Middle Jurassic and Late Cretaceous sedimentary rocks, and the Late Cretaceous of the Mahajanga Basin has been explored via collaborative expeditions between United States and Malagasy universities [36], revealing a diverse terrestrial fauna. India and Madagascar are the smallest continents by land area: at approximately 3.9 million square kilometres, they are an order of magnitude smaller than Africa (approx. 30.37 million square kilometres) and are therefore likely to have fewer fossils. Nonetheless, the potential for finding new dinosaurs among India’s under-explored Mesozoic outcrops seems high.

Many more dinosaurs are known from Africa than from Antarctica, Australia or India and Madagascar but, aside from a few regions, the continent is generally poorly sampled (figure 2). Dinosaurs are well known from the Late Jurassic of Tanzania [18,37] and the Late Triassic and Early Jurassic of South Africa [38–40] but have not been intensively studied elsewhere, despite numerous reported occurrences. Dinosaur body fossils are known from the Early and Middle Jurassic and Late Cretaceous of Morocco [41–44], the Late Triassic of Zimbabwe [45], the Middle Jurassic and Early Cretaceous of Niger [46,47], the Early Cretaceous of Algeria [48] and Malawi [49], and the Late Cretaceous of Egypt [50–52], to give some examples. In many of these places, the climate is semi-arid, providing excellent outcrop. However, with the exception of Egypt and South Africa, many African countries have limited vertebrate palaeontological resources, and in some cases, political conditions have constrained expeditions; for example, the UK’s Foreign, Commonwealth and Development Office currently advises against all travel to Algeria, Niger and Libya. The prospects for finding new dinosaurs in many parts of Africa, however, appear to be excellent if political conditions allow.

## 5. What time periods represent the ‘frontier’ for new dinosaur exploration?

**Figure 3.**
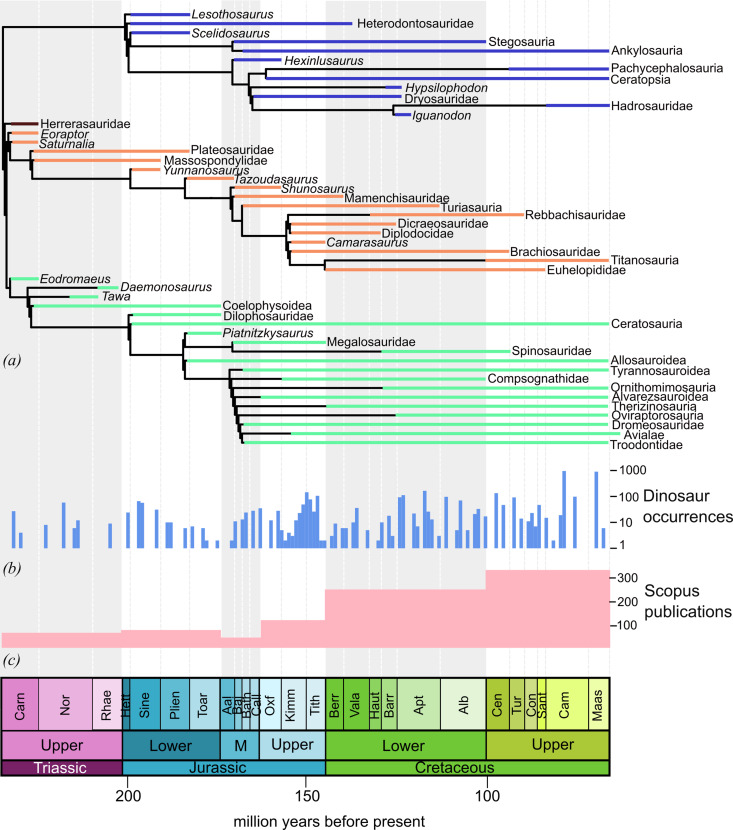
(*a*) Time-calibrated consensus dinosaur phylogeny. Coloured bars indicate taxon ranges. Blue bars are ornithischians, orange are sauropodomorphs and green are theropods. (*b*) Dinosaur occurrences in 1 million-year time bins through the Mesozoic on a logarithmic scale. (*c*) Number of publications on Scopus over the past 10 years on dinosaurs from each time period.

Figure 3 shows a time-calibrated consensus phylogeny of dinosaurian relationships, along with two curves that are proxies for sampling effort. One is a curve of number of dinosaur (including avialan) occurrences (data from the Paleobiology Database, paleobiodb.org, downloaded 22 January 2025), while the other is the number of publications referencing specific geological periods and dinosaur fossils from 2014 to 2023.

The time-calibrated phylogeny indicates that the most significant times of major clade diversification are (i) the Carnian, with the split of Sauropodomorpha, Theropoda and Ornithischia, (ii) the Triassic–Jurassic boundary, with the origin of early diverging ornithischian clades, Sauropodiformes and Neotheropoda and (iii) the Middle Jurassic, when almost all of the major dinosaurian clades were established and radiated. Subsequent to these three time periods, there were important diversifications *within* clades, such as the origin of hadrosaurs within ornithopods, but most of the well-established major branches of the dinosaur evolutionary tree were established prior to the Late Jurassic [53].

### (a) The Carnian

The earliest unequivocal dinosaurs are Carnian in age, and they appear to have diversified immediately after the Carnian Pluvial Episode [54], a time of major environmental change, marine extinctions and biotic radiations [55] linked to the eruption of the Wrangellia Large Igneous Province [56,57]. Records of dinosaurs prior to the Carnian (e.g. *Nyasasaurus* [58]) are both highly fragmentary and poorly dated [59]. Despite a long history of research on Carnian dinosaurs, there is still much uncertainty around the phylogenetic positions of many taxa (recently reviewed in [60]), and even the relationships between the major clades themselves are the subject of a heated debate [61–63]. *Pisanosaurus mertii* may be a basal ornithischian [64], or a non-dinosaurian silesaurid [65], or silesaurids themselves may be basal ornithischians [66]. The identification of silesaurids as a paraphyletic assemblage of early diverging ornithischians [66] would explain approximately 30 million-year ghost lineage at the base of Ornithischia (figure 3*a*) but is not currently widely accepted. Herrerasaurids could be basal theropods, basal saurischians or could be the sister taxon of Theropoda [60,61,64,67]. Theropoda and Ornithischia could be more closely related to each other than either is to Sauropodomorpha (the Ornithoscelida hypothesis [61]), or Sauropoda and Theropoda could be sister taxa to the exclusion of Ornithischia (the Saurischia hypothesis [63]). Only through new, more complete fossil discoveries in the Carnian (and perhaps also older strata) will we be able to address these questions. The Late Triassic is uniformly poorly sampled (figure 3*b*), and there were five times more publications on Late Cretaceous dinosaurs than there were on Late Triassic dinosaurs over the past 10 years (figure 3*c*). This is likely to be, at least in part, due to the increasingly poorly preserved fossil record in older rocks, as well as dinosaurs being genuinely less abundant components of ecosystems than in later epochs.

### (b) The Triassic–Jurassic boundary

The Triassic–Jurassic boundary marks one of the ‘big five’ mass extinctions of the Phanerozoic: the end-Triassic mass extinction (ETME [68]). Widely considered to have been caused by major climate perturbations linked to the eruptions of the Central Atlantic Magmatic Province [69], the ETME caused major extinctions in both the marine and terrestrial realms [68]. Following the ETME, dinosaurs diversified and increased in abundance [70,71], and some analyses have suggested that the Early Jurassic was the most important time period for their diversification [53]. The sampling curves (figure 3*b*,*c*) show relatively large numbers of occurrences immediately following the ETME. Understanding of the impact of the extinction on dinosaurs is limited by poor temporal constraints for many fossiliferous terrestrial formations on either side of the boundary. Highly temporally constrained studies of latest Triassic and earliest Jurassic ecosystems and new fossil discoveries are needed.

### (c) The Middle Jurassic to Oxfordian

During this time, the major ornithischian lineages Thyreophora, Ornithopoda and Marginocephalia diverged from one another and diversified, the macronarian, diplodocid, dicraeosaurid, rebbachisaurid and turisaurian sauropod clades were established, the megalosauroids and all coelurosaurian clades branched from one another and birds originated. Diversifications at this time have been linked to the Jenkyns Event [72], a time of major climatic change caused by the eruption of the Karoo–Ferrar Large Igneous Province in the Toarcian that caused the Toarcian Oceanic Anoxic Event in the oceans and a major hyperthermal on land [73]. Some clades that are modelled to have originated at this time currently lack a Jurassic fossil record (e.g. Therizinosauria, Oviraptorosauria; figure 3*a*), but the timing of their diversification can be established based on the recognition of dromaeosaurid and troodontid teeth [74] and probable avialan footprints [75] in the Middle Jurassic, indicating substantial ghost lineages. The sampling curves show that the Middle Jurassic has few recorded dinosaur occurrences, especially in the early part (figure 3*b*), and there have been fewer publications on dinosaurs from this time period than any other during the Mesozoic (figure 3*c*) over the last decade. Limited research focus on the Middle Jurassic, at least historically, may be due to a lack of Middle Jurassic terrestrial rocks across Europe and North America due to high sea levels at this time, despite the irony that the first dinosaur to be named, *Megalosaurus*, was found in Middle Jurassic rocks [1].

## 6. Future prospects for dinosaur discovery, taxonomy and systematics

Understanding evolution, extinction and their drivers through time is a central tenet of palaeobiology. In order to address major outstanding questions about Mesozoic ecosystems, we need to find new fossils that represent new taxa and more complete fossils of existing taxa, and we need to be able to place them accurately in time. The above review of the ‘frontier’ of dinosaur exploration in both time and space indicates that Carnian, Triassic–Jurassic boundary and Middle Jurassic rocks offer excellent potential for new discoveries that could fundamentally change our understanding of the evolution of the dinosaurs. Moreover, Gondwanan continents, especially Africa and India, have great potential to yield surprising new discoveries in not only these time intervals but also across their entire Mesozoic history, as shown by many exciting recent discoveries such as *Spicomellus* [42], *Chilesaurus* [76] and *Jakapil* [77].

Accurately constraining the age of new and existing discoveries is of vital importance in order to understand the context of dinosaur evolution and extinction. It is becoming increasingly recognized that studies that attempt to examine global patterns of diversity change (e.g. [78,79]) have neither the temporal nor spatial resolution to achieve their aims and are beset by uneven sampling biases that strongly affect their results [80–83]. Regional studies, with high levels of temporal resolution and more even sampling biases, are required to accurately reconstruct biodiversity patterns in ecologically meaningful ways [82]. Large advances in our understanding of faunas have been obtained from regional stratigraphic studies using a combination of approaches such as radiometric dating, magnetostratigraphy, chemostratigraphy and sequence stratigraphy to accurately date quarries (e.g. [6,84–89]) and, in the future, increasing collaborations between field palaeontologists and stratigraphers are needed.

A challenge to the future of dinosaur discovery is funding. The willingness of national-level public funding agencies to support palaeontological fieldwork is variable, but in at least some countries, such as the UK, funding panels are often risk averse and less likely to fund prospecting for and excavation of fossil vertebrates because of the potential that expeditions may prove unsuccessful and the long timescales often involved in preparing and studying specimens after collection (RJ Butler 2024, personal observation). The funding gap is partially filled by grant schemes from smaller charities and professional associations (e.g. small grant schemes from the Palaeontological Association and Society of Vertebrate Paleontology), but these often provide small-scale resources that are not sufficient to support large multi-year fieldwork programmes that are necessary for discovering dinosaur fossils (a sauropod skeleton, even once discovered, may take weeks to months to excavate). Among a substantial number of other factors, the funding gap has led to huge declines in academic-led dinosaur excavation in some countries, such as the UK. Similarly, taxonomic research, especially beyond the description of new species, is often seen as unglamorous and lacking in impact and is, as a result, challenging to fund.

The challenges of funding fieldwork and taxonomy are not unique to palaeontology but have been recognized in other fields, such as ecology and zoology [90,91]. In ecology, the term ‘extinction of experience’ has been used to refer to the progressive loss of direct human interactions with nature [91], with a broad range of negative consequences. We see the potential for a similar ‘extinction of experience’ within the palaeontological community, as ever greater numbers of students and researchers have limited or no experience of or training in the basic field- and specimen-based research that underpins the subject. To address this, it is vital that we emphasize the critical importance of fieldwork, taxonomy and systematics to the field and continue to provide basic training to undergraduate and graduate students in these areas.

One ‘solution’ that some palaeontologists have chosen to take to overcome the challenges of funding fieldwork is to effectively outsource this by purchasing fossils from commercial collectors (e.g. [92,93]). In some cases—such as the commercially operated Solnhofen quarries in Germany or the Jehol Group fossils in China where many discoveries are made by local farmers—purchasing fossils may be the most or only feasible approach [94,95]. However, there are numerous potential drawbacks. Commercially collected fossils may have limited or no geological data, limiting understanding of their precise stratigraphic history, palaeoenvironmental setting or taphonomic history. They may be ethically problematic, lacking appropriate collections permissions and in some cases having been illegally exported [96,97]. Moreover, there is a thriving industry in producing modified or fake fossils, which can be sufficiently compelling as to convince experienced palaeontologists [98–100]. Extreme caution is, therefore, warranted if purchasing commercially collected fossils that they are ethical, well provenanced and unmodified, and it is far more desirable in most contexts for palaeontologists to lead their own field expeditions where fossils can be collected with full documentation and context.

Dinosaur research is sometimes mentioned in the context of concerns around ‘parachute science’, where researchers from high-income countries (typically in the Global North) conduct work, especially field-based studies, in low-income countries with little or no engagement from local researchers or communities. Several key examples are often cited. One is the Brazilian theropod dinosaur ‘*Ubirajara jubatus*’, described by a team of non-Brazilian researchers based on an illegally exported specimen in a German museum that was subsequently repatriated to Brazil after a high-profile campaign for its return (e.g. [96,97]). Another is an exceptionally preserved specimen of the dinosaur *Psittacosaurus*, smuggled out of China and currently held in a museum in Germany [96]. Examples such as these are typically linked to smuggling of privately collected fossils for commercial sale rather than to academic fieldwork led by museums or universities. Although examples undoubtedly exist, we currently lack well-documented cases of modern academic dinosaur fieldwork as ‘parachute science’.

In contrast, there are plentiful examples of genuine collaborations with researchers and communities local to the investigated sites, alongside scientific capacity-building, public engagement and benefit-sharing through infrastructure development or geotourism, with collected fossils typically being reposited permanently in appropriate public collections within their country of origin. Examples include the Qhemegha project in South Africa [101], the Madagascar Paleontology Project (https://www.dmns.org/science/research/madagascar-paleontology-project/) and projects on Cretaceous vertebrates from Sudan [102]. The research field would benefit from better documentation and visibility of these kinds of important projects—how they are established, funded, developed and their impacts—to facilitate sharing of best practice and the development of important case studies of the societal and scientific benefits of collaborative international fieldwork.

## 7. What techniques might change how we search for new dinosaurs in the future?

There is considerable current interest in how artificial intelligence and machine learning might be applied in biological taxonomy. Although there are significant challenges given the morphological complexity of vertebrate skeletons and taphonomic variation in fossil preservation, machine learning approaches have already been used to successfully taxonomically classify dinosaur teeth [74,103,104] and tracks [105,106]. The use of such approaches in taxonomic studies is likely to grow in the near future [107].

Dinosaur taxonomy has traditionally been based upon the external morphology of the preserved skeletal and dental elements. Soft tissue is too infrequently preserved to be of substantial use to date in dinosaur taxonomy, although the growing understanding of scale and feather morphology within dinosaurs could lead to greater use for taxonomy in the future [108]. Likewise, the ever-increasing understanding of dinosaur bone histology and advances in molecular palaeontology (e.g. [109]) might provide new approaches to distinguishing species. However, given the known rate of decay of DNA, it is unlikely that this will ever be used as a tool to distinguish fossil dinosaur species or reconstruct phylogenies [110]. Despite this, molecular palaeontology has much to offer dinosaur palaeobiology. The increasing sensitivity of instruments that are able to identify fossil biomolecules and the validation of methods that allow tissue-specific signals to be distinguished despite thermal alteration during burial [111] allow reconstruction of attributes like physiology and metabolism [112] that would have been unthinkable just a decade ago.

In many aspects, the methods used to discover and collect dinosaur fossils have changed little since the nineteenth century. Discovery typically still involves time-intensive manual ‘prospecting’, involving teams walking large distances of often remote and challenging terrain, with a high level of serendipity and risk of no or limited finds [9]. Collection involves excavation with hand tools and plaster jacketing. However, new technologies have provided opportunities to enhance the chance of successful outcomes. Geographical information systems modelling and remote sensing approaches have been developed to predict the distribution of fossil-bearing localities, potentially reducing prospecting area and time [113–115], although such approaches require further development and are most applicable to areas already known to yield fossils rather than completely new areas previously unprospected.

Another potentially promising approach to shortening prospecting time is the use of remote sensing and/or drones to image potentially promising areas and identify fossil bone [116–118], although such techniques remain very much in their infancy. LIDAR (light detection and ranging) and photogrammetry, increasingly available through standard smartphones, also have potential relevance for the three-dimensional recording of quarry data during excavations of dinosaur fossils [119–121].

## 8. Conclusion

Taxonomy and systematics underpin all aspects of palaeobiology, and it is vital that their importance is recognized by funders, institutions and publishers, and that we continue to train early career researchers in these disciplines to avoid an ‘extinction of experience’ in specimen- and field-based research. Much of the responsibility for this must sit with palaeontologists themselves, as it is they that typically form editorial boards, sit on grant funding panels, review funding proposals and manuscripts and decide how to train students. Despite more than 200 years of exploration and discovery of dinosaurs, there are still many questions left to address. Many of these require the discovery of new taxa and more complete fossils of existing taxa, while others might be answered by reappraisal of existing specimens in the light of new data or via the application of new techniques. The Carnian, Triassic–Jurassic boundary and Middle Jurassic time intervals, and new finds in Gondwana, especially Africa and India, offer the best opportunities to make major new discoveries that could fundamentally change our understanding of dinosaur evolution. The application of remote sensing and drone imaging to help us narrow down the best areas to prospect, three-dimensional scanning to record fossils in the field and in the laboratory, and artificial intelligence and machine-learning applied to help identify problematic fossils could revolutionize the field in the future.

## 9. Methods

### (a) Collector curves

All dinosaur regular genera and species, both valid and invalid, were downloaded from the Paleobiology Database (PBDB; paleobiodb.org) on 17 December 2024. The data were cleansed to remove Avialae, ichnotaxa and ootaxa. Taxa that were listed as invalid due to misspellings, obsolete variates or that were renamed for grammatical or linguistic reasons were removed. *Nomina dubia, nomina nuda,* objective and subjective synonyms and recombinations were retained. Collector curves (figure 1) were built in R 3.4.0 [122]. Code and raw data are available in [123].

### (b) Time-calibrated phylogeny

A consensus dinosaur phylogeny was manually produced in Mesquite [124]. First and last appearance data were collected for all taxa in the phylogeny and are listed in the data file provided in [123]. First and last appearances generally correspond to the earliest and latest dates of the Stage from which the taxon is known, unless more accurate information was available in the literature. Ages were derived from the International Chronostratigraphic Chart v. 2024/12. The consensus phylogeny was time-calibrated using the TimePalaeoPhy function in the R package Paleotree [125] with minimum branch lengths of 0.5 million years. Code and raw data are available in [123].

### (c) Occurrences through time histogram

All dinosaur body fossil occurrences were downloaded from the PBDB on 22 January 2025 and were manually cleansed to remove ichnotaxa and ootaxa. The midpoint of the first appearance datum and last appearance datum was taken for each occurrence, and these midpoints were plotted in 1 million year bins in R 3.4.0 [122]. Code and raw data are available in [123].

### (d) Occurrences map

Non-avialan dinosaur occurrences, excluding form taxa, were downloaded from the PBDB (27 January 2025). These were manipulated in R 4.4.2. The dataset was pruned to limit it to species type occurrences only. Triassic, Jurassic and Cretaceous occurrences were plotted onto modern day maps using the packages *maps* and *ggplot*. Code and raw data are available in [123].

### (e) Publications through time

Data on trends in taxonomic publications were derived from the abstract and citation database Scopus (accessed 8 March 2025). Two primary searches were conducted of a decade of recent publications (2014−2023): (i) a keyword search for ‘dinosaur AND taxonom*’, with the wildcard operator used to ensure that both ‘taxonomy’ and ‘taxonomic’ were included; (ii) a keyword search for ‘dinosaur’. Publication sets were then exported to SciVal, which enables trends (e.g. by author country) to be examined and citation metrics (e.g. CiteScore) to be calculated for groups of publications. Summaries of the data generated by these searches are provided as [123]. Scopus was also used to determine the number of publications for Mesozoic epochs using keyword searches in the format of ‘dinosaur AND (“Late Cretaceous” OR “Upper Cretaceous”)” for the time interval 2014−2023 (see figure 3).

## Data Availability

All code and raw data are available from the Dryad Digital Repository [123].
